# Urinary extracellular vesicles miRNA—A new era of prostate cancer biomarkers

**DOI:** 10.3389/fgene.2023.1065757

**Published:** 2023-01-20

**Authors:** Garima Jain, Parimal Das, Prashant Ranjan, Neha, Ferran Valderrama, Clara Cieza-Borrella

**Affiliations:** ^1^ Centre for Genetic Disorders, Institute of Science, Banaras Hindu University, Varanasi, India; ^2^ Centre for Biomedical Education, Cell Biology and Genetics Research Centre, St. George’s University of London, London, United Kingdom

**Keywords:** prostate cancer, biomarker, micro-RNA, liquid biopsy, extracellular vesicles (EV)

## Abstract

Prostate cancer is the second most common male cancer worldwide showing the highest rates of incidence in Western Europe. Although the measurement of serum prostate-specific antigen levels is the current gold standard in PCa diagnosis, PSA-based screening is not considered a reliable diagnosis and prognosis tool due to its lower sensitivity and poor predictive score which lead to a 22%–43% overdiagnosis, unnecessary biopsies, and over-treatment. These major limitations along with the heterogeneous nature of the disease have made PCa a very unappreciative subject for diagnostics, resulting in poor patient management; thus, it urges to identify and validate new reliable PCa biomarkers that can provide accurate information in regard to disease diagnosis and prognosis. Researchers have explored the analysis of microRNAs (miRNAs), messenger RNAs (mRNAs), small proteins, genomic rearrangements, and gene expression in body fluids and non-solid tissues in search of lesser invasive yet efficient PCa biomarkers. Although the presence of miRNAs in body fluids like blood, urine, and saliva initially sparked great interest among the scientific community; their potential use as liquid biopsy biomarkers in PCa is still at a very nascent stage with respect to other well-established diagnostics and prognosis tools. Up to date, numerous studies have been conducted in search of PCa miRNA-based biomarkers in whole blood or blood serum; however, only a few studies have investigated their presence in urine samples of which less than two tens involve the detection of miRNAs in extracellular vesicles isolated from urine. In addition, there exists some discrepancy around the identification of miRNAs in PCa urine samples due to the diversity of the urine fractions that can be targeted for analysis such as urine circulating cells, cell-free fractions, and exosomes. In this review, we aim to discuss research output from the most recent studies involving the analysis of urinary EVs for the identification of miRNA-based PCa-specific biomarkers.

## 1 Introduction

Prostate cancer (PCa) is the most common type of male cancer and one of the leading causes of male cancer mortality worldwide (Roehrborn and Black, 2011; Siegel et al., 2021). Advances in clinical screening and early detection of the disease through the detection of prostate-specific antigen (PSA) have resulted in a 90% 5-year survival rate; yet, increase the risk for a man to face a diagnosis of PCa even when the tumor is benign or low-risk (Martin et al., 2018). The United States Preventive Services Task Force has also advised against PSA screening due to its inability to distinguish high-risk PCa and to the high numbers of false positive results that this test provides (Fenton et al., 2018). Therefore, it is necessary to identify PCa-specific biomarkers that allow not only for an early and accurate disease diagnosis but also for tumor stratification in order to provide the best patient clinical management.

### 1.1 Current status of diagnostics in PCa and the need for new biomarkers

PCa diagnosis currently relies on digital rectal examination (DRE) and blood PSA levels followed by a solid biopsy to confirm and characterize the malignancy. DRE sensitivity and specificity are reported to be 80% and 48.6% respectively which makes it dependent on validation *via* solid biopsy upon palpation and detection of irregularities in the prostate nodules (Irekpita et al., 2020). Although DRE provides significantly higher success rates in detecting high-grade PCa cases when compared to PSA levels, it still shows a limited overall predictive value and high invasiveness (Jones et al., 2018).

In spite of the recent major developments in biochemical tests and imaging techniques, the primary biochemical tool for PCa diagnosis is serum PSA levels. PSA-based screening is a non-invasive and painless technique that has contributed to reducing the incidence of advanced disease and mortality improving overall PCa diagnosis and prognosis (Martin et al., 2018). However, it is worth mentioning that PSA reliability as a sole biomarker thus remains controversial due to the high rate of false-positive results and low specificity (Fenton et al., 2018).

PSA is a prostate-specific serine protease produced by the prostate epithelium whose function is to liquefy the seminal coagulum allowing sperm to swim freely (Balk et al., 2003). In normal health conditions, epithelial cells release PSA to the seminal fluid and only a small amount escapes into blood circulation through an unknown molecular mechanism (Stenman et al., 1999). Apart from cancer, other pathological conditions such as benign prostatic hyperplasia (BPH) and prostatitis are normally associated with increased PSA levels in the blood (Gao et al., 2019). On this note, the European Randomized Study of Screening for Prostate Cancer has reported that 22%–43% of the PSA-detected malignancies are overdiagnosed (Hugosson et al., 2019) which leads to unnecessary biopsies and over-treatment. In the absence of alternative biochemical PCa biomarkers, the majority of PCa screening guidelines still advise the measurement of total serum PSA (tumor (t)PSA>4 ng/mL) as a benchmark marker for the detection of PCa. However, in the last few years novel PCa detection assays, such as the 4K score and Prostate Health Index (PHI) which integrate PSA with other biomarkers (Fossati et al., 2015; Parekh et al., 2015), have emerged with the aim of reducing the rates of false positives and the number of unnecessary biopsies (Table 1). Nevertheless, these approaches still involve the validation of the results through the extraction of solid biopsies (Voigt et al., 2017).

**TABLE 1:** List of commercially available clinical tests used for PCa diagnosis and confirmation.

Test name^a^	Biomarker	Nature of the material analyzed	Source of biomarker	Outcome	References
Pro-PSA/PHI	Pro-PSA/PHI	Protein	Blood serum	distinguish between benign prostatitis and PCa	Fossati et al. (2015)
4K score	Total PSA, free PSA, intact PSA, and human kallikrein 2	Protein	Blood serum	predict the risk of having a Gleason Score >7	Parekh et al. (2015)
SelectMDx	DLX1 and HOXC6	mRNA	Urine	Detect likelihood of detecting PCa upon biopsy	Van Neste et al. (2016)
Confirm MDX	DNA hypermethylation	DNA	Tissue biopsy	Evaluate the need of re-biopsy in case of inconclusive biopsy	Stewart et al. (2013)
PCA3	PCA3 gene expression	mRNA	Urine	Distinguish between benign and PCa	Hessels et al. (2003)
ExoDx	Expression of ERG and PCA3	mRNA	Urinary exosomes	Validates the need for biopsy	Margolis et al. (2022), Salagierski and Schalken (2012)

^a^ Test names are the commercialized brand-names for the tests; Pro-PSA, precursor of PSA; PHI, prostate health index; PSA, prostate specific antigen; DLX1, distal-less homeobox 1 and HOXC6, Homeobox C6; PCA3, prostate cancer antigen 3; ERG, ETS-related gene.

In the last few years, biopsy-based approaches have been notably improved and highlighting guided biopsy techniques such as Transrectal ultrasound scan (TRUS)-guided biopsy, which have been developed with the goal of reducing procedural mistakes (Hodge et al., 1989). Despite the significant technological advances, investigations so far have demonstrated that guided biopsy still exhibits a significant prevalence of false negatives and tumor under grading due to imprecise positioning of the biopsy needle when compared to radical prostatectomy-informed grading (Wolters et al., 2010).

Thus, there is a clear need for identifying more accurate and reliable, and less invasive PCa biomarkers that overcome the limitations associated with PSA tests and guided biopsy techniques. Additionally, new biomarkers should ideally help improve treatment decision-making through more accurate detection of minimal residual disease and a more efficient clinical staging of the disease taking into account the complexity and heterogeneous nature of PCa at the different stages (i.e., localized tumor, metastatic, and hormone-independent recurrence of PCa). Data so far suggest that novel biomarkers should not be considered as mutually exclusive parameters; instead, their effectiveness should be assessed in conjunction with the current approaches and decision-making algorithms (Fossati et al., 2015; Parekh et al., 2015).

### 1.2 A new generation of diagnostic biomarkers in PCa

Over the last few decades, a new generation of PCa prognostics and diagnostics tests have emerged. Each test combines several parameters and biomarkers and provides a specific utility and timing of usage in line with the disease stages; for instance, PHI and 4K-score tests are used for diagnostics purposes (Fossati et al., 2015; Parekh et al., 2015), Oncotype DX is applied in risk identification (Hessels et al., 2003; Covas Moschovas et al., 2022), and Polaris and Decipher are used for active monitoring and management of PCa cases (Banerjee and Punnen, 2022). In Table 1 we can observe a summary of the currently available PCa diagnostic tests, apart from PSA and histopathological analysis, used in patients with suspected or proven PCa using molecular markers derived from solid tissue, blood, or urine.

The list of commercialized tests stated in Table 1 shows that mRNAs (PCA3, ExoDx, SelectMDx) and protein expression (PHI and 4K Score) analyzed in liquid biopsies such as blood serum and urine are being extensively used as potential alternatives to DRE, PSA, and solid biopsies. The detection of miRNAs in liquid biopsies for PCa diagnostic, prognosis, and staging in it is still in the early research stage; up to date, there are no commercialized diagnostic kits that involve the detection of miRNAs in PCa or any other type of cancer disease.

### 1.3 Urine components used for the identification of biomarkers

Urine is considered a rich source of biomarkers for malignancies related to the urinary tract; since it contains cellular debris, proteins from glomerular filtration, and secretions of the urogenital tract, it can reflect a person’s real-time pathophysiology of a disease. For instance, expressed prostatic secretion (EPS) is a fluid released by the prostate to the urine through the urethra, following a digital rectal prostate massage (Drake et al., 2009); it is rich in proteins and metabolites that could provide meaningful information on prostate health status (Drago et al., 2021). However, different urine fractions has been targeted for the identification of new biomarkers like miRNA (e.g., urinary circulating cells, cell-free DNA, and EV) and this, together with the variations in urine collection times, sample sizes, miRNA isolation protocols, the number of miRNAs analyzed, and the internal control employed, is responsible for the discrepancies noted in the PCa-related urine miRNAs reported in the literature to date.

### 1.4 EV miRNAs as biomarkers

EVs are lipid-bound vesicles that originate from the endosomal system or bud off from the plasma membrane and that contain miRNAs that are transported to target cells for intercellular communication. Prostate cell-derived EVs can be released into the urine through the urethra where they are detected by simple non-invasive techniques. EVs include exosomes (30–100 nm), prostasomes (50–500 nm), estosomes (50–1,000 nm), oncosomes (50–500 nm) and microvesicles (100–1,000 nm) (Drake et al., 2009). It has been demonstrated that EVs mediate cell-cell communication by carrying active genetic material such as miRNAs and mRNAs (Akers et al., 2013; Maia et al., 2018). Since miRNAs are highly stable inside the EVs, easily available body fluids such as urine, blood, semen, and breast milk, have been investigated for the detection and analysis of extravesicular miRNAs that can inform about healthy and pathological conditions (Lässer, 2013; Gámez-Valero et al., 2015; Groot and Lee, 2020). Given that cancer cells are reported to abundantly shed EVs (Brassart et al., 2019), it is possible to detect tumor miRNAs within EVs that inform cancer cell activity (Lässer, 2013; Cheng et al., 2014).

However, one of the main limitations of working with extracellular miRNAs is the restricted available published data on the optimal conditions for their isolation and analysis and even more limited in regard to prostate cell-derived extravesicular miRNAs.

Thus, we aim here to review the PCa-specific studies involving the detection of urine-derived EVs-associated miRNAs in order to develop a reliable miRNA panel that can provide information about disease diagnosis, stage, and progression.

## 2 Materials and methods

### 2.1 Literature search

Using the electronic database PubMed, we performed a review of studies published before July 2022 that involved the analysis of urine-derived EV-miRNAs in PCa diagnosis. Potentially relevant studies were identified using the PubMed string search and Boolean operators: (“urine”[Subheading] OR “urine”[Text Word] OR “urine”[MeSH Terms]) OR (“urinary tract”[MeSH Terms] OR “urinary tract”[Text Word]) AND (“microRNAs”[MeSH Terms] OR “microRNAs” [Text Word] OR “mirna”[Text Word]) AND (“prostatic neoplasms”[MeSH Terms] OR “prostate cancer”[Text Word]); AND (“exosome”[MeSH Terms] OR “microvesicles”[Text Word] OR “extracellular vesicles”[Text Word]).

### 2.2 Data extraction

Only the articles that fulfilled all the eligibility criteria, as below, were selected:

(1) Research articles that presented the diagnostic potential of urinary miRNAs in PCa (Siegel et al., 2021); studies that used only uEV (exosomes, microvesicles) as sources to identify miRNAs (Martin et al., 2018), studies that showed data related to the detection of miRNAs focused only in urine or in combination with tissue, serum, plasma, or cell line. Exclusion criteria were as follows (Roehrborn and Black, 2011): case reports, editorials, reviews, duplication of selected articles, and retracted articles (Siegel et al., 2021); studies that report methods and/or protocols.

We excluded non-English studies, studies using miRNAs from cell-free urine fractions, and studies dated before 2007.

The following information was collected from the selected articles and tabulated in Tables 2, 3 (Roehrborn and Black, 2011). Authors’ name, and publication year (Siegel et al., 2021); type of study design—case-control or cohort (Martin et al., 2018); sample size—number of benign, PCa, and healthy controls (Fenton et al., 2018); the volume of urine used for extraction and method of EV isolation (Irekpita et al., 2020); urine collection time point (pre- or post-DRE or DRE not considered) (Jones et al., 2018); method of miRNAs shortlisting for validation, type of RNA extraction (Balk et al., 2003); normalization of miRNA quantification (Stenman et al., 1999); differentially expressed miRNA reported (Gao et al., 2019); identified miRNA after validation, and (Hugosson et al., 2019) it is predictive value as compared to PSA (Tables 2, 3).

**TABLE 2:** Summary of relevant studies and urinary-extracellular vesicles miRNAs that they have proposed as potential biomarkers for PCa diagnosis, prognosis and risk classification.

Study	Country of origin/Population of study	Control population	Normalizer miRNA	Relevant outcome	miRNA reported	Predictive value of study compared to PSA test
Bryzgunova et al. (2016)	Russia	Healthy males (age-matched not defined)	miR-16	Differentiating PCa patients from healthy individuals	miR-19b	Not compared
Samsonov et al. (2016)	Russia	healthy males, age-matched, without clinical PC-relevant manifestations, PSA level below 4 ng/mL	U6 snRNA and hsa-miR-191-5p	Differentiating PCa patients from healthy individuals	miR-574-3p, miR-141-5p, and miR-21-5p	Not compared
Foj et al. (2017)	Spain	healthy males, negative DRE, PSA level below 4 ng/mL	spike-in control cel-miR-39	Differentiating between healthy subjects and low-risk PCa group versus intermediate and high-risk PCa	miR-21 and miR-375	Not compared with PSA. Found to be associated with Gleason grade
Koppers-Lalic et al. (2016)	Netherland	Patients with negative biopsy, abnormal DRE, elevated PSA level	Not mentioned	Differentiating patients (high PSA) who actually need biopsy	isomiRs of miR‐21, miR‐375 and miR‐204 present more than mature miRNA	ROC improved when PSA is combined with miRNA variant
Wani et al. (2017)	India	BPH	Not mentioned	Differentiating A. BPH from prostate cancer and, B. prostate cancer from bladder cancer	A. miR-615-3p	Not mentioned
					B. miR-2909	
Rodríguez et al. (2017)	Norway	Healthy, age-matched males	Average of three miRNAs/small RNAs; miR- 10b-5p, let-7b-5p and U6 snRNA	Differentiating PCa patients from healthy individuals	miR-196a-5p and miR-501-3p	Not mentioned
Xu et al. (2017)	China	Healthy, age-matched males and BPH	Spike-in control cel-miR-39	Differentiating PCa from BPH.	miR-145	miR-145 combined with serum PSA performs better than PSA or miRNA alone
Hasegawa et al. (2018)	United States	Healthy donors	U6 snRNA	Differentiating high-grade PCa from low-grade, implied to be used for RNA treatment purpose	miR-888 and miR-891a	Not mentioned
Lekchnov et al. (2018)	Russia	Healthy donors	Normalized using the pair ratio method	A. Healthy versus cancer	A. miR-16.5p miR-24.3p miR-30b.5p	Diagnostic performance of miRNA combinations was found to be better than PSA alone
				B. Healthy versus benign	B. miR-31.5p miR-660.5p miR-107	
					miR-30e.3p miR-29a.3p	
				C. Benign versus cancer	C. miR-191.5p miR-22.3p	
Fredsøe et al. (2019)	Denmark	patients with radical prostatectomy	Average of miR-200b-3p, miR-27b-3p and miR-30b-5p	Logistic regression model Predicting recurrence-free survival	miR-151a-5p, miR-204-5p, miR-222-3p, miR-23b-3p and miR-331-3p along with PSA	diagnostic performance of miRNA combinations was found to be better than PSA alone
Bryzgunova et al. (2019)	Russia	Healthy, age matched males	Ratio based normalization	miRNA panel based on classifier to differentiate between healthy, BPH and PCa group	5 miRNA pairs (miR-30a: miR-125b; miR-425: miR- 331; miR-29b: miR-21; miR-191: miR-200a; miR-331: miR-106b)	Better performance reported for paired miRNA as compare to individual miRNA or PSA
Matsuzaki et al. (2021)	Japan	Healthy donors with a serum PSA below 4 ng/mL	KLK3 gene	miRNA panel based on classifier to differentiate between healthy, PCa group	miR-30b-3p and miR-126-3p	AUC better than that of PSA
Konoshenko et al. (2020)	Russia	Healthy donors and BPH	Spike-in control cel-miR-39	Differentiate between healthy, BPH, and PCa group	Combination of miR-19b, miR-30e, miR-31, miR-92a, miR-125, miR-200, miR-205, and miR-660	Not mentioned
Davey et al. (2020)	Canada	BPH	SNORD44	Differentiate BPH and PCa group	A combination of miR-375 and miR-574	Not compared with PSA
Li et al. (2021)	China	Healthy age matched	Spike-in control cel-miR-39	Differentiate metastatic and localized PCa group	miR-375	AUC of miRNA is superior to PSA
Kim et al. (2021)	Korea	Non-recurrent cancer	RNU6B	Identify biochemical recurrence of PCa	miR‐26a‐5p, miR‐532‐5p, and miR‐99b‐3p	Not applicable
Ruiz-Plazas et al. (2021)	Spain	Patients treated by radical prostatectomy	arithmetic mean of hsa-miR-423-5p, SNORD38B, SNORD49A, hsa-miR-191-5p, hsa-miR-103a-3p and U6 small nuclear RNA	Prediction of PCa severity (ISUP classification of low risk and high risk PCa)	miR-221-3p, miR-222-3p	Reported miRNA combined with sTWEAK shows higher accuracy using only serum PSA levels
Holdmann et al. (2022)	Germany	BPH	NGS based quantification	differentiating PCa from BPH.	miR-6749-5p and miR-532-3p along with 8 miRNA panel	Not applicable

**TABLE 3:** Summary of studies involving the analysis of urinary-extracellular vesicle (EV) miRNAs that have been analyzed in this review including information on the urine volume used for the isolation of EV, the isolation method, the method used for EV miRNA extraction, and the approach followed to select the miRNAs of study.

Study	Urine volume	Post-DRE collection	Extracellular vesicles isolation method	RNA extraction method	miRNA selection/detection method
Bryzgunova et al. (2016)	13 mL	Not Mentioned	Ultracentrifugation added with filtration = microvesicles, and exosomes	DNA or RNA isolation kit (Biosilica Ltd., Russia)	Selected miRNA from previous studies
Samsonov et al. (2016)	40 mL—lectin agglutination method, 13 mL—centrifugation method	Before DRE	Differential centrifugation and lectin induced precipitation	RNA isolation Kit (BioSilica, Russia)	Selected miRNA from previous studies
Foj et al. (2017)	30–50	Post DRE	Differential centrifugation	miRNeasy serum/plasma kit (Qiagen)	Shortlisted five cancer-associated miRNAs
Koppers-Lalic et al. (2016)	20–90 mL	Post DRE	Differential centrifugation	Trizol LS	Small RNA sequencing
Wani et al. (2017)	Not mentioned	Not mentioned	Exiqon miRCURYTMexosome isolation	miRNeasy mini kit	Selected from previous study
Rodríguez et al. (2017)	Not mentioned	Not mentioned	sequential centrifugation	mirCury, miRNeasy and trizol	NGS
Xu et al. (2017)	200 mL	Not mentioned	hydrostatic filtration dialysis, ultracentrifugation	Spiked with Caeno-rhabditis elegans miR 39 (cel-miR-39) (TIANGEN) when incubation in Trizol	Selected miRNA from previous studies
Hasegawa et al. (2018)	Not mentioned	Not mentioned	filter and ultracentrifuged	mirVana miRNA Isolation Kit (Ambion)	Entire miR-888 cluster (based on previous study)
Lekchnov et al. (2018)	Not mentioned	Not mentioned	Ultracentrifugation	Denaturing of exosomes followed by Na-acetate precipitation, purified using spin column	Selected from previous studies
Fredsøe et al. (2019)	Not mentioned	Before DRE	miRCURY Exosome Isolation Kit	miRCURYTM RNA Isolation Kit	miRNAs previously found consistently detectable in urine from PC patients
Bryzgunova et al. (2019)	20–30 mL	Not mentioned	Ultracentrifugation	Phenol chloroform	miRNA from a pre-formed Urine Exosomes Focus Panel (Exiqon, Denmark)
Matsuzaki et al. (2021)	38.5 mL	Post DRE	Ultracentrifugation	miRNeasy Mini Kit (Qiagen, Venlo, Netherlands), Clean-up Kit (Qiagen), and RNA MS2 (Roche Diagnostics, Mannheim, Germany)	Microarray
Konoshenko et al. (2020)	5 mL	Not mentioned	Ultracentrifugation	Gu/OcA miRNA isolation	miRNA biomarkers of PCa from previous study
Davey et al. (2020)	5 mL	Post DRE	Vn96 Peptide Based isolation	RNeasy Plus Micro Kit (QIAGEN, Valencia, CA, United States)	miRNA from bio fluid of PCa patient from previous study
Li et al. (2021)	10 mL	Before DRE	ExoQuick-TC for tissue culture media and urine (System Biosciences)	miRNeasy Serum/Plasma kit (Qiagen, Hilden, Germany	NGS
Kim et al. (2021)	10 mL	Catheterized during radical prostectomy	ATPS (Exo2D, EsosomePlus, Seoul, South Korea)	miRNeasy Serum/Plasma Kit (Qiagen, Hilden, Germany	NGS
Ruiz-Plazas et al. (2021)	Not mentioned	Post DRE	ExoRNeasy Kit (Qiagen)	ExoRNeasy Kit (Qiagen)	Cell line based selection
Holdmann et al. (2022)	4 mL	Not Mentioned	Norgen Biotek Urine exosome isolation kit	Norgen Biotek Urine exosome isolation kit	NGS

### 2.3 Identification of prostate-specific miRNA-target gene

TargetScan and miRDB were used to extract the list of genes that are the most commonly reported as target genes for the particular miRNAs. Only the target genes with a score of >95 were taken into account. Using the term “Prostate cancer” in the gene-cards database, a different list of 12,400 genes related to prostate biology was retrieved. Items in both lists were then compared to find common elements, and the results were plotted in a Venn Diagram using Venny2.1 (Supplementary Material).

## 3 Results

### 3.1 Summary of studies included

Following the inclusion criteria, a total of 74 primary articles were found in Pubmed from which 38 were discarded as they were duplicated studies, methodology studies, or reviews. After applying the exclusion criteria, a final number of 18 worldwide studies were selected for this review (Tables 2, 3). Seventeen of the study designs were case-control. The sample size ranged between 14 and 619 per study with a total of 1750 samples analyzed overall. The volume of the urine sample analyzed across studies ranged from 5 to 90 mL.

### 3.2 Documentation of findings

We notice a considerable degree of inconsistency in the identified biomarkers if we solely concentrate on research that involves uEVs-related miRNAs. Interestingly, not only the differential expression patterns but also the differences in the miRNA distribution pattern, and the existence of miRNA isomeric forms have been crucial parameters for the identification of new PCa biomarkers. Outcomes of the studies that investigated miRNA levels in uEVs included but were not limited to, early diagnosis, differentiating BPH from cancer, predicting biochemical recurrence, and anti-miRNA-based treatment.

The miRNAs that were assessed and identified as relevant biomarkers varied widely (Table 2). Since miRNAs have a variety of functions and strongly-correlated target genes, it would be more logical to evaluate frequently reported PCa biomarker miRNAs based on their prostate-specific target genes and functions; thus we have focused on the target gene evaluation of a total of eight miRNAs that were reported in more than one of the studies included in this review: miR-19b, miR–21-5p, miR-141-5p, miR-375, miR-331-3p, miR-10b-5p, miR-204, and miR-30a/30b (Bryzgunova et al., 2016; Koppers-Lalic et al., 2016; Samsonov et al., 2016; Foj et al., 2017; Bryzgunova et al., 2019; Fredsøe et al., 2019; Matsuzaki et al., 2021). All the eight selected miRNAs, except miR-10b-5p, showed >50% of target genes as PCa-associated genes (Supplementary Data; Table 4).

**TABLE 4:** Most frequently reported dysregulated miRNAs in urinary exosomes of prostate cancer patients. The list of targeted genes for each miRNA was retrieved using miRDB and TargetScan. Only the target genes with a score of >95 were considered in the next step. Another list of prostate-associated genes was retrieved using the keyword “Prostate cancer” in gene cards, providing the output of around 12,400 genes. Elements on both lists were then compared to retrieve common elements using Venny2.1 software and the output result of the common element was collected in the form of a Venn Diagram and list.

S.N.	miRNA	Number of total miRNA target gene elements	Number of common element	% Of miRNA target genes that are PCa associated	Name of miRNA target genes that are PCa associated
1	miR-21	24	12	50	FASLG, PBRM1, SKP2, PLAG1, VCL, RBPJ, TGFBI, SPRY1, GATAD2B, ADGRG2, KRIT1, FGF18
2	miR-141-5p	10	5	50	EGFR, DLC1, MAP3K1, MAP3K7CL, NUP50
3	miR-331-3p	4	3	75	NRP2, PHLPP1, PTPN2
4	miR-19b	148	87	58	PIK3CA, ESR1, TSC1, PMEPA1, IGFBP3, PSAP, BMPR2, TRIM33, PIK3CB, MDM4, SULF1, ZNF217, CCNL1, DDX3X, ACSL4, WNK1, ATF2, PIK3R3, CSMD1, SATB1, ABCA1, DLG5, SKIL, CLOCK, RICTOR, HBP1, LDLR, MAP3K12, RNF11, PTPRD, MECP2, AKAP1, TFCP2L1, TNRC6B, SGK1, SPOCK1, RORA, PPP1R12A, ITGB8, DNAJA2, LRP2, CACNA1C, S1PR1, RPS6KA5, CEP350, KHDC4, KIF13A, LRIG3, SHCBP1, EDARADD, ELOVL5, TSHZ3, E2F8, CNTFR, SOX6, CACUL1, BMP3, UBE2D2, DDX6, DTNA, USP33, STOX2, ADRB1, MBNL2, SPATA2, MRTFB, SIN3B, SIPA1L1, ATG14, EPS15, ACOX3, ACBD5, ARAP2, SLC6A8, NAV3, KCNJ2, ATXN1, ATXN7L1, ANKIB1, ZMYND11, ATP6V1B2, ABR, AFF1, GRSF1, RAP2C, ZBTB4, KPNA6
5	miR-375	4	3	75	ELAVL4, RLF, POC1B
6	miR-204	71	49	69	FBXW7, SIRT1, NR3C1, ANGPT1, ACSL4, FOXC1, SOX11, NBR1, EPHB6, DLG5, DMTF1, ESRRG, PTPRD, SH3PXD2A, MMP16, TNRC6B, FRS2, GCNT2, KLF12, WWC3, ITPR1, RAB10, RPS6KA5, IL7R, FBN2, CREB5, RIOK1, PRRX1, ACER3, KXD1, CDK13, EBF2, SPRED1, TCF12, TMOD3, NCOA7, C9orf72, HOOK3, CNOT1, RHOBTB3, NBEA, C2orf68, SLC37A3, SOX14, PID1, B3GNT5, EVC2, AP2A2, PHOX2B
7	miR-10b-5p	10	4	40	RORA, E2F7, CADM2, SOBP
8	miR-30a/30b	215	118	54	VIM, ITGA6, SOX9, NFIB, HDAC9, NCAM1, SOCS3, RUNX2, SOCS1, CYP24A1, PRUNE2, ELK1, MTDH, CHD1, ARID2, CNOT9, ELL2, XPO1, PTPN13, FAP, RARG, MYH11, LIN28B, EML4, NT5E, FRZB, CHL1, SIX1, ZMYND8, NEDD4, SMAD1, DLG5, RUNX1, CCNE2, CLOCK, SCARA5, PTP4A1, AZIN1, RASA2, KLF10, SPEN, SH3PXD2A, TBL1XR1, TNRC6B, CALCR, RORA, EED, BDP1, ANKRD17, KLF12, SH2B3, RFX6, UBE2V2, PCDH17, PPARGC1B, PNKD, FOXG1, SCN9A, CBX5, TNRC6A, E2F7, PLAGL2, TP53INP1, XPR1, ANKHD1, SRSF7, DDAH1, CFL2, BRD1, SLC35C1, STK39, ACTR3C, ADAMTS9, SNX16, ADAM19, PON2, EEA1, SEC23A, NFAT5, GNL3L, IRGQ, GALNT7, RGS8, WDR82, PRDM1, PTGFRN, CCDC97, STOX2, RTKN2, PFN2, TWF1, USP37, SETD5, REEP3, LIMCH1, C9orf72, HOOK3, APOBEC3F, LCLAT1, BRWD3, SLC12A6, PLEKHO2, PIP4K2A, SLC35A3, GMNC, TMEM170B, NAV3, SNX18, YPEL2, RFX7, ADRA2A, RAP2C, B3GNT5, STK35, COL13A1, PPP3R1, EML1

## 4 Discussion

### 4.1 Discussion on potential PCa miRNAs biomarkers based on their prostate-specific target-genes

#### 4.1.1 miR-21-5p

Elevated expression of miR-21 has been reported in PCa tissue (Damodaran et al., 2021), plasma (Bryant et al., 2012), and urine (Samsonov et al., 2016). miR-21 is known to regulate the proliferation and apoptosis of cancer cells through the PTEN/PI3K/AKT pathway which makes it a good potential novel target for anti-cancer therapies (Liu et al., 2019). In Samsonov et al. (2016) the analysis of PCa–related miRNAs using lectin-induced agglutination method for isolation of in urinary-exosomes revealed significant upregulation of miR-21-5p in PCa patients with PSA ranging from 10 to 20 as compared to healthy control. Although the study reported a notable diagnostic significance of miR-21-5p along with miRNA-574-3p and miR-141-5p, the sensitivity of miR-21-5p (area under the curve (AUC) and receiver operating characteristic curve (ROC) 0.65; 95%, CI = 0.477–0.814, sensitivity = 0.46, *p* < 0.05) was the lowest among the three. Another RNAseq study performed by Koppers-Lalic et al. (2016) on PCa patients (PSA = 8.5–9.5) observed a significant upregulation of miR-21 expression (*p* = 0.02) along with miR-375, and miR-204 as compared to healthy controlKoppers-Lalic et al. (2016). When the expression of these miRNAs was validated by RT-qPCR, the results regarding miR-21 were not significant; however, further studies showed later that those differences were associated with the presence of different miR-21-5p isomeric forms. This study proposed that the detection of isomiRs in combination with PSA levels could significantly increase the probability of detecting PCa as compared to mature miRNA or PSA alone (Koppers-Lalic et al., 2016).

Many studies have proposed the establishment and analysis of batteries of miRNAs, instead of individual miRNA, in order to achieve higher sensitivity and specificity in the diagnosis and stratification of PCa patients (Bryzgunova et al., 2019; Fredsøe et al., 2019; Davey et al., 2020). For instance, a study conducted by Bryzgunova et al. (2019) in uEVs highlighted the relevance of miR-21-5p in the discrimination of PCa and BPH patients when they applied an algorithm-based classifier, which analyzed the expression of five different pairs of diagnostically significant miRNAs: miR-21-5p+miR-29b, miR-30a+miR-125b, miR-425+miR-331, miR-191+miR-200a and miR-331+miR-106b.

To corroborate the association of miR-21-5p with PCa, in this review we have cross-checked the group of miR-21-5p target genes with the PCa-associated genes retrieved from GeneCards, resulting in a list of common genes shown in Table 4. The top 12 miR-21-5p target genes showing a score >95 are PCa-specific genes. This result, together with the aforementioned findings, highlights the relevant role of miR-21-5p in urine-based PCa diagnosis and underlines the necessity of conducting functional studies that help us identify the efficacy and suitability of miR-21-5 and its isomiR, alone or in combination with PSA or other miRNAs, in PCa diagnosis.

#### 4.1.2 miR-141-5p

miR-141 is reported to affect angiogenesis, proliferation, and metastasis in colon cancer and nasopharyngeal carcinoma (Dong et al., 2019; Tong et al., 2021). Although its role in PCa is yet to be defined, studies have reported miR-141 to be commonly present in the blood of PCa patients (Yaman Agaoglu et al., 2011). In regard to studies performed in urine samples, Xu et al. (2017) did not find any significant differences in the expression of uEV miR-141-5p between BPH and PCa but its presence in urinary cells of PCa patients was observed to be significantly higher in the disease group. In contrast, other authors like Samsonov et al. (2016) found upregulated expression of uEV miR-141-5p in PCa patients when compared with healthy donors.

When we cross-checked the miRNA-141-5p target genes with the PCa-specific genes, we observed that the top five common genes are mainly involved in cell survival which does not shed enough light on the role of this miRNA in PCa disease progression (Supplementary Data; Table 4).

To summarize, the role of miR-41-5p as a PCa-specific biomarker remains questionable due to the low statistical significance of the results obtained in the referred studies; research involving larger case-control cohorts is required to obtain concluding results.

#### 4.1.3 miR-375-3p

There exist contradicting findings in regard to this miRNA. Published data shows increased miR-375 levels in the blood of PCa patients with metastatic disease (Bhagirath et al., 2020). Additionally, a Next-Generation Sequencing (NGS)-based investigation revealed that miR-375-3p is significantly downregulated in PCa patients and combination of miR-375 and miR-451a has the potential to serve as a specific and sensitive molecular marker to differentiate PCa patients from BPH patients, and that miR-375 alone can distinguish localized PCa patients from metastatic PCa (Li et al., 2021). However, there is still disagreement about the putative discriminating function of miR-375 in PCa. In a study conducted by Foj et al. (2017), it was demonstrated that both miR-375 and miR-21 are significantly upregulated in intermediate and high risk PCa patients as copare to low risk and healthy population and could discriminate between PCa and non-PCa participants that had shown high PSA levels. Although, another study observed downregulation of miR-375-5p in cancer cases with NGS data from uEVs, the mature versions of miR-375, -141, and -21 cannot effectively distinguish between PCa and healthy individuals unlike their isomeric forms (Koppers-Lalic et al., 2016). In summary, the presence of miR-375 in uEVs has notably been observed in studies, but the significance of mature miR-375 as an overexpressed biomarker is debatable.

#### 4.1.4 miR-10b-5p

NGS of uEVs has revealed miR-10b-5p and other miRNAs such as let-7b-5p, miR-30a-5p, miR-10a-5p, and let-7a-5p, as the most abundant in urinary exosomes (Cheng et al., 2014; Koppers-Lalic et al., 2016; Rodríguez et al., 2017). Their presence does not seem to be associated with the disease state and, although further investigation is required, it has been suggested that these miRNAs could be applied as uEVs-specific normalizers. In fact, one of the studies used an average of three miRNAs/small RNAs for normalization (miR-10b-5p, let-7b-5p, and U6 snRNA) as miR-10b-5p and let-7b-5p were expressed similarly in the control and patient groups according to the NGS study (Rodríguez et al., 2017).

#### 4.1.5 miR-331-3p

miR-331-3p has been reported to affect long-term survival in hepatocellular cancer (Chang et al., 2014). It is also a mediator in the epithelial-to-mesenchymal transition in PCa (Fujii et al., 2016) which has led to its consideration as PCa prognostic marker as it is also supported by its identified target genes in Table 4.

miR-331-3p has already been included in large cohort-based case-control studies as part of a predictor panel for biochemical recurrence and aggressive PCa risk (Fredsøe et al., 2019). Also, one study involving the use of RT-qPCR to quantify miRNAs in urine supernatant observed that miR-331-3p together with miR-92a allowed the differentiation of BPH and PCa cases from healthy controls with a 52% sensitivity (*p* = 0.025) (Bryzgunova et al., 2019). The same study also analyzed the expression values of miR-331-3p in uEVs which were paired up with miR-425; in that case, the algorithm could differentiate PCa from healthy and BPH cases with a 40% sensitivity (*p* = 0.002). The combination of miR331-3p and miR-106b in urine supernatant and microvesicles was also evaluated and could differentiate PCa from healthy and BPH cases with a 30% sensitivity (*p* = 0.036). This study concluded that a five-miRNA pairs panel could be used to develop an expression-based algorithm that allows us to classify PCa, BPH, and healthy with 100% specificity and 97.5% accuracy (Martin et al., 2018).

#### 4.1.6 miR-19b

The presence of miR-19b in uEVs has been proposed as an auxiliary PCa differentiating criterion with a 100% specificity and a 93% sensitivity (Bryzgunova et al., 2016). High levels of this miRNA have been found in both blood serum of PCa patients and mice prostate tumors tissue which supports a potential diagnostic and a prognostic value (Duca et al., 2021). One study conducted by Konoshenko et al. (2020) included miR-19b in a battery of twelve miRNAs that were investigated in 31 combination ratios. Eight of those miRNAs in six ratios, including miR-19b/miR-92a in uEVs could discriminate PCa, BPH, and healthy donors with 100% specificity, and 100% sensitivity. Overall, studies so far suggest a key role of miR-19b, alone or in combination with other miRNAs, in the differentiation of healthy vs. PCa as well as BPH vs. PCa. Moreover, in this review, miR-19b has shown the highest number of prostate-associated target genes with a total of 84 genes (Table 4).

#### 4.1.7 miR-204

miR-204 is known to be an NF-kB pathway inhibitor and therefore hinders various tumor progression-related phenomena including metastasis (Todorova et al., 2016; Wa et al., 2019). The relevance of miR-204 in PCa has been confirmed in studies using RT-qPCR (Fredsøe et al., 2019) and NGS (Koppers-Lalic et al., 2016). Fredsøe et al. (2019) performed a novel logistic regression model comprising PSA and five urine miRNAs, one of them miR-204, which could significantly predict biochemical recurrence and clinical risk stratification of PCa. But further research on this miRNA has demonstrated that not only the copy number variation of miR-204 in uEVs but also the presence of its different isoforms (isomiRs) with 3′-end modifications have the ability to discriminate PCa cases from the healthy subjects. In this sense, a study conducted by Koppers-Lalic et al. (2016) observed that standard mapping protocols involving expression levels of matured miRNAs failed to robustly discriminate disease status whereas isomiRs of miR-21, miR-204, and miR-375 could detect PCa with 72.9% sensitivity and 88% specificity, with an AUC of 0.866.

#### 4.1.8 miR-30a/30b

The miRNA-30 (miR-30) family is a group of tumor suppressor miRNAs consisting of six mature miRNA molecules (miR-30a, miR-30b, miR-30c-1, miR-30c-2, miR-30d, and miR-30e) (Mao et al., 2018). miR-30a has been frequently reported to be downregulated in PCa and its expression inhibits androgen-independent growth in PCa (Zhao et al., 2019). In this sense, deep sequencing analysis conducted by Rodríguez et al. (2017) described miR-30a-5p as one of the most abundant miRNAs in uEVs slightly, but not significantly, downregulated in PCa cases compared to healthy males. In the aforementioned study involving the application of a five-miRNA pair-based algorithm, miR-30a was one of the miRNAs that could discriminate between PCa and BPH patients and healthy samples with 100% specificity and 97.5% accuracy (Bryzgunova et al., 2019). Since miR-30b is also among the most stably expressed miRNAs in uEVs along with miR-200b-3p, and miR-27b-3p, it has been used as an endogenous control in RT-qPCR experiments in some studies (Fredsøe et al., 2019) which questions its validity as PCa biomarker. Nevertheless, in another study, miR-30b-3p along with miR-126-3p was observed to be overexpressed in uEVs of PCa patients as compared to subjects showing cancer-negative biopsy results. This study proposed a PSA and age-adjusted logistic regression analysis for the prediction of PCa with 46.4% sensitivity and 88.0% specificity (Matsuzaki et al., 2021). miR30b along with miR-30e also showed up as members of the most diagnostically significant miRNA pairs in an algorithm created to differentiate PCa patients from a healthy population. This study used median ∆Ct difference, and confidence interval as statistical criteria for determining reliable markers of PCa (Lekchnov et al., 2018).

Finally, there are other miRNAs that have been reported to show significantly low expression levels in PCa compared to a healthy population and these are miR-196a-5p, miR-34a-5p, miR-143-3p, miR-501-3p, and miR-92a-1-5p in uEVs (Rodríguez et al., 2017). According to one study, miR-2909 can be used to monitor PCa aggression (Wani et al., 2017). Another study observed elevated levels of miR-888 cluster in patients with high-grade prostate cancer as compared to lower grades of the disease (Hasegawa et al., 2018). Three miRNAs from uEVS - miR-26a-5p, miR-532-5p, and miR-99b-3p, are suggested to be used in the prediction of biochemical recurrence of PCa (Kim et al., 2021). Using a machine learning method, the two miRNAs hsa-miR-532-3p and hsa-miR-6749-5p could distinguish BPH patients from those with PCa with 80% specificity and 66.7% sensitivity (Holdmann et al., 2022). miR-423-5p and miR-193-3p were downregulated in uEVS of high-risk patients’ post-digital rectal examination but their diagnostic significance is not known (Ruiz-Plazas et al., 2021).

### 4.2 Limitations and potential cofounders

The observations reported for the majority of the aforementioned uEV miRNAs show inconsistent or contradictory results despite the fact that few of them are common to the 18 studies included in this review: miR-21-5p, miR-375, miR-125, miR-19b, miR-191, miR-200, miR-30b, miR-574-3p (Table 5). The origin of this inconsistency could reside in the lack of consensus in the volume of urine used for the study, in the heterogeneity of methodology applied for the isolation of both uEVs and miRNAs, and in the purification of these nucleic acids (Table 2); nonetheless, we have also observed high variability in other aspects related to the selection of subjects and miRNAs for study and analysis of results.

**TABLE 5:** Proposed miRNAs panel with potential use for the discrimination between control or bening prostatic hyperplasia populations and prostate cancer patients. Using each miRNA as an array element, the table shows the reporting frequency for each miRNA and the studies where they were reported.

Item	Frequency occurrences	References of studies
miR-21-5p	4	(Samsonov et al., 2016)
		Foj et al. (2017)
		Koppers-Lalic et al. (2016)
		Bryzgunova et al. (2019)
miR-375	3	Foj et al. (2017)
		Koppers-Lalic et al. (2016)
		Davey et al. (2020)
miR-125	2	Bryzgunova et al. (2019)
		Konoshenko et al. (2020)
miR-19b	2	Bryzgunova et al. (2016)
		Konoshenko et al. (2020)
miR-191	2	Lekchnov et al. (2018)
		Bryzgunova et al. (2019)
miR-200	2	Bryzgunova et al. (2019)
		Konoshenko et al. (2020)
miR-30b	2	Lekchnov et al. (2018)
		Matsuzaki et al. (2021)
miR-574-3p	2	Samsonov et al. (2016)
		Davey et al. (2020)

Next, we will expand on these study limitations and potential cofounders that may be influencing the associations observed between the miRNAs and PCa diagnosis, prognosis, and stratification.

#### 4.2.1 Sample-selection cofounders

**FIGURE 1:**
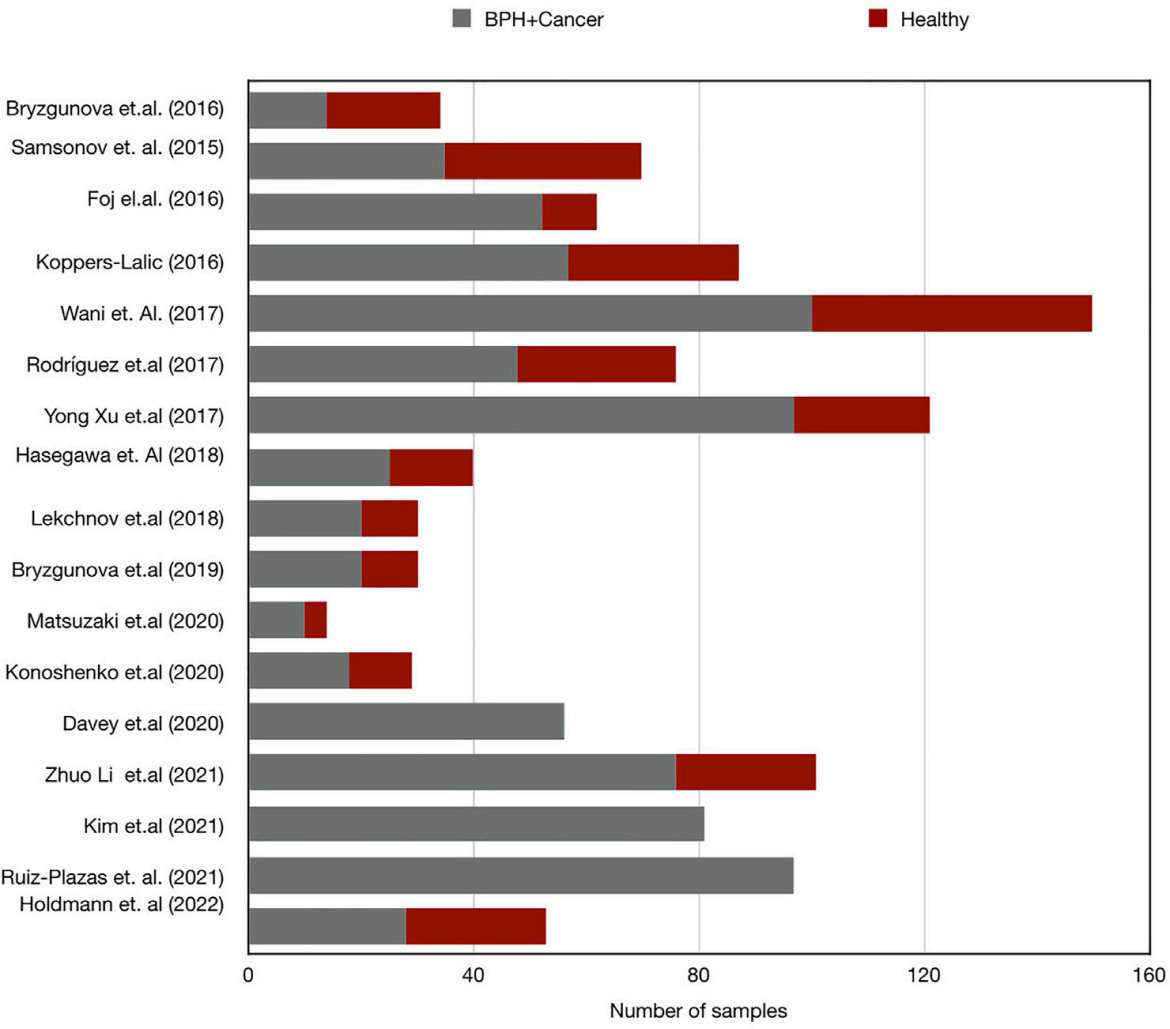
Bar chart demonstrating the number of samples in case and control group sets in miRNA research in PCa. Control, healthy subject; BPH, Benign prostate hyperplasia; Cancer, prostate cancer.

We believe that the disparity in the results could be influenced by the variations in the selected control groups; whilst ten of the studies included in this review involved healthy donors as controls (Bryant et al., 2012; Cheng et al., 2014; Bryzgunova et al., 2016; Fujii et al., 2016; Samsonov et al., 2016; Wani et al., 2017; Bryzgunova et al., 2019; Dong et al., 2019; Fredsøe et al., 2019; Liu et al., 2019), three studies compared PCa with BPH samples (Foj et al., 2017; Damodaran et al., 2021; Kim et al., 2021), two studies looked at both BPH and healthy controls (Koppers-Lalic et al., 2016; Davey et al., 2020), and one study used non-recurrent PCa as control (Hasegawa et al., 2018) (Figure 1; Table 3).

Studies involving healthy subjects as control groups were subject to diversity of the population characteristics or previous history of infections which, together, may bias the miRNA-level differences between groups. With the exception of a few studies where controls were mentioned to be age-matched (Bryant et al., 2012; Cheng et al., 2014; Bryzgunova et al., 2016; Fujii et al., 2016; Koppers-Lalic et al., 2016; Liu et al., 2019), most of the studies did not mention age or risk factors in the matched controls. Furthermore, the inclusion of BPH patients as a control population adds an extra layer of diversity to the global picture.

In regard to the case group, all studies rightly disclosed detailed information on the patient population analyzed including PSA values, Gleason scores, and clinical presentation (Table 2).

#### 4.2.2 Diversity in sample collection and processing

The collection of urine samples for prostate-related analysis can take place before/pre-DRE or after/post-DRE. Prostate stimulation during a DRE generates the release of cells and vesicles to the urethra, thus urine collection post-DRE is advised for the identification of PCa biomarkers (Pellegrini et al., 2017). Five publications in our review (Koppers-Lalic et al., 2016; Foj et al., 2017; Davey et al., 2020; Matsuzaki et al., 2021; Ruiz-Plazas et al., 2021) highlighted post-DRE urine sample collection whereas three have done so pre-DRE (Samsonov et al., 2016; Fredsøe et al., 2019; Li et al., 2021) and the others have not specified this criterion. Barreiro et al. (2021) have demonstrated that storage time, temperature, and format impact the quantity and quality of the uEV miRNAs yielded. Most of the studies reviewed did not detail the sample storage conditions and those that did, indicated a storage temperature of −80° (Koppers-Lalic et al., 2016; Samsonov et al., 2016; Xu et al., 2017; Bryzgunova et al., 2019; Fredsøe et al., 2019; Davey et al., 2020; Kim et al., 2021; Li et al., 2021; Matsuzaki et al., 2021); other authors preferred to use fresh samples in order to avoid miRNAs degradation or loss. We believe that these aspects should be taken into account in future experiments and their standardization would help decrease inconsistencies in the results obtained across studies.

#### 4.2.3 Selection of miRNAs, methods of expression quantification and analysis

Another limitation identified in this review is the variability in the criteria used for the initial selection of the miRNAs of study; only few studies employed initial microarrays (Bryzgunova et al., 2019; Matsuzaki et al., 2021) or deep sequencing techniques for their further validation in case-control populations (Rodríguez et al., 2017; Kim et al., 2021; Li et al., 2021; Holdmann et al., 2022) whereas the rest of the projects based their miRNA selection on previously published data reporting a correlation between their high levels of expression in serum/blood/tissue and PCa.

In regard to the endogenous controls used in the miRNAs quantification, some studies utilized spike-in miRNA levels like cel-miR-39 (Foj et al., 2017; Xu et al., 2017; Konoshenko et al., 2020; Li et al., 2021), and others used more than one endogenous miRNA or their arithmetic mean (Rodríguez et al., 2017; Fredsøe et al., 2019; Ruiz-Plazas et al., 2021) and others utilized the pair ratio method (Lekchnov et al., 2018; Bryzgunova et al., 2019). In this sense, this field lacks validated internal controls and, given that the exact mechanisms of action of many miRNAs are yet to be elucidated, we may be using controls whose function is associated with PCa disease which could provide unreliable results.

The statistical approaches used in the analysis of the expression of uEV miRNAs evaluated by RT-qPCR also differed across studies. For instance, early studies in the field used the Mann–Whitney test and T-test (Bryzgunova et al., 2016; Samsonov et al., 2016; Foj et al., 2017; Wani et al., 2017; Xu et al., 2017; Hasegawa et al., 2018; Lekchnov et al., 2018) to determine the significance of the identified miRNAs in PCa diagnosis whereas most recent studies applied uni- or multivariate regression-based methods (Fredsøe et al., 2019; Davey et al., 2020; Li et al., 2021).

### 4.3 Future studies

While urine-derived EVs-associated miRNA biomarkers have shown promising results in the early detection and stratification of PCa, there is no current evidence for their application in the screening or diagnosis of the disease in clinical practice until their clinical significance is further validated. The field of liquid biopsies is expected to evolve in the next few years toward the standardization of the isolation and analysis methods; moreover, technological advances involving the development of more sensitive and specific uEV binding resins will also help mitigate the methodological limitations associated with urine EVs purification. miRNAs, single or in combination with other miRNAs or with other PCa diagnostic tools such as PSA and imaging techniques, are promising strategies yet to refine. On this note, future studies investigating the potential diagnostics value of uEV miRNAs should involve a minimum of a 1-year patient follow-up in order to be able to adequately assess the functionality of the reported biomarker. The approaches for miRNA screening and selection should be unbiased and broader, involving deep sequencing of urine miRNAs that provides a complete miRNA profile in liquid biopsy; as discussed in this review, only a few studies have been close to achieving these goals. The selection of only previously-described PCa miRNA markers for further analysis limits the results and does not open any option to the identification of new biomarkers; this is substantially relevance given that this is a relatively new field lacking further information and knowledge on the biological mechanisms of miRNA. In addition, to avoid measurement bias, all the studies should consider stringent quality checks during sample processing and miRNA measurements such as staged non-human spiked in miRNA and the inclusion of biological and experimental replicates. They should also take into account potential confounders including patients’ risk factors and medications. This review has also detected a lack of consensus in the scientific community in regard to the urine sampling timing, pre- or post-DRE, and the sample volume. Another important point needing improvement in order to ensure data reproducibility is the use of both a discovery and a validation cohort; this approach will help in identifying significantly dysregulated microRNAs in a discovery cohort followed by validation of these findings in an independent larger validation cohort.

### 4.4 Review limitations

This review did not apply any quality assessment tool like QUADAS for diagnostic accuracy studies, as it was not feasible due to the heterogeneity of the data. For this review, we only selected papers that have been published in the English language.

## 5 Conclusion

Taken together, the results of the studies covered in this review suggest that the application of uEVs miRNAs in PCa prognostics, diagnostic, and stratification could be effective in combination with traditional tools such as PSA and imaging. There are several limiting factors in the studies published so far involving uEV miRNAs and PCa in regard to the analysis of small case-control populations, the inconsistencies in sample collection times and processing, the limited patient follow-up over time, and the criteria used for miRNA selection.

In this review, we have considered studies on uEV miRNAs that differentiated the control population from BPH subjects and compared them with PCa patients. Based on the collected information, we propose here a panel of eight miRNAs, including miR-21-5p, miR-375, miR-125, miR-19b, miR-191, miR-200, miR-30b, and miR-574-3p, to further investigate and apply in PCa diagnosis, prognosis and stratification.

In the near future, the application of standardized protocols along with the development of multicentre studies involving large cohorts of patients and controls will allow us to define and validate the role of the uEV miRNAs as PCa biomarkers that can be applied in clinical practice.
